# Temporal Trends and Short‐ and Long‐Term Mortality of People With Acute Myocardial Infarction and Rheumatoid Arthritis: A Nationwide Cohort Study

**DOI:** 10.1002/acr.70009

**Published:** 2026-03-07

**Authors:** Megan Butler, Nicholas Weight, Muhammad Rashid, Rodrigo Bagur, Purvi Parwani, Samantha Hider, Edward Roddy, Robert Butler, Mamas A. Mamas

**Affiliations:** ^1^ Keele University Keele United Kingdom; ^2^ University of Leicester Leicester United Kingdom; ^3^ National Institute for Health Research Leicester Cardiovascular Biomedical Research Unit, Glenfield Hospital Leicester United Kingdom; ^4^ Western University London Ontario Canada; ^5^ Loma Linda University Health Loma Linda California; ^6^ Midlands Partnership Foundation Trust Stoke‐on‐Trent United Kingdom; ^7^ Royal Stoke University Hospital, University Hospitals North Midlands Stoke‐On‐Trent United Kingdom; ^8^ National Institute for Health and Care Research Birmingham Biomedical Research Centre Birmingham United Kingdom

## Abstract

**Objective:**

We investigated whether a diagnosis of rheumatoid arthritis (RA) affects the quality of inpatient acute myocardial infarction (AMI) care and long‐term mortality post‐AMI.

**Methods:**

We analyzed data from 784,091 adults, 6,047 with a diagnosis of RA, from England and Wales hospitalized with AMI between 2005 and 2019 from the Myocardial Ischaemia National Audit Project registry, linked with Office for National Statistics mortality data and hospital episode statistics. Cox regression models were used to compare risk of all‐cause mortality at different time points according to the presence of RA.

**Results:**

There was no difference in adjusted 30‐day mortality between groups (adjusted hazard ratio [aHR] 1.09, 95% confidence interval [CI] 0.99–1.19; *P* = 0.075). Beyond this, at 1 year (aHR 1.14, 95% CI 1.07–1.21), 5 years, (aHR 1.28, 95% CI 1.23–1.33), and to the end of the study period (aHR 1.31, 95% CI 1.26–1.36), the risk of all‐cause mortality was significantly higher in patients with RA (all *P* < 0.001). Risk of cardiovascular mortality was not significantly different at 30 days or 1 year (aHR 1.08, 95% CI 1.00–1.17; *P* = 0.058), but it was at 5 years (aHR 1.15, 95% CI 1.08–1.23; *P* < 0.001) and to the study endpoint post‐AMI (aHR 1.18, 95% CI 1.11–1.24; *P* < 0.001).

**Conclusion:**

We found no meaningful disparities in inpatient care according to the presence of RA; however, those with RA have elevated long‐term all‐cause mortality post‐AMI. Our findings suggest that the mortality burden of RA post‐AMI is not driven by the quality of AMI care during admission and is likely driven by the progressive nature of the comorbidities and the complications of treatments associated with RA.

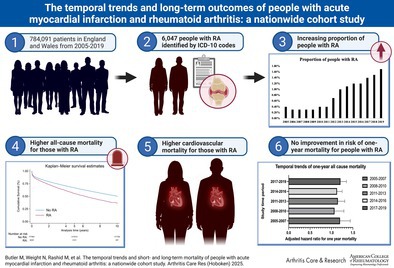

## INTRODUCTION

Rheumatoid arthritis (RA) is part of a heterogeneous group of autoimmune rheumatic diseases, affecting approximately 1% of the world's population, and is associated with progressive disability and premature death.^1^ Importantly, cardiovascular disease (CVD), including acute myocardial infarction (AMI) is the most common cause of mortality in patients with RA.^2^

**SIGNIFICANCE & INNOVATIONS**

- In a nationwide cohort study of 784,091 patients with acute myocardial infarction (AMI) between 2005 and 2019, patients with rheumatoid arthritis (RA) received the same standard of care as patients without RA.
- Patients with RA had elevated long‐term all‐cause mortality, but 30‐day mortality post‐AMI was not significantly higher than that of patients without RA.
- There is a long‐term elevated risk of cardiovascular‐specific mortality, which manifests at 5 years and beyond.

People with RA are at elevated risk of AMI,^3^ with the systemic proinflammatory state of RA driving accelerated atherogenesis,^4^, ^5^ and at increased risk of long‐term mortality following AMI, consistently shown across different health care settings.^6^, ^7^ The impact of RA on in‐hospital outcomes and care quality is more complex. US data have suggested lower in‐hospital mortality and comparable quality of care after AMI,^8^ whereas similar studies from Australia have shown poorer in‐hospital care and outcomes for those with RA.^9^ RA disease severity is correlated with poorer long‐term mortality, especially higher glucocorticoid doses,^7^ with higher coronary plaque burden shown in people with poorer RA control.^10^ However, there are encouraging data suggesting a potential reduction in major adverse CV events (MACEs) and reduced vascular inflammation among individuals with RA treated successfully with immunomodulatory therapy.^11^, ^12^

We used data from the Myocardial Ischaemia National Audit Project (MINAP) registry, linked to Office for National Statistics (ONS) mortality data and hospital episode statistics (HES), to investigate the in‐hospital quality of care that patients receive according to RA status and to assess whether this influences their long‐term all‐cause and CV mortality over an extended period of follow‐up for a median duration of >6 years and whether mortality risk has changed over our study period.

## METHODS

### Study design

This study was a retrospective cohort study of routinely collected data from MINAP linked to the ONS and HES. MINAP is a prospective national registry of patients with acute coronary syndrome admitted to 230 hospitals in the United Kingdom. MINAP includes data on patient demographics, clinical characteristics, comorbidities, pharmacotherapy, management, and in‐hospital outcomes on approximately 90,000 patients admitted with acute coronary syndrome per year. The ONS is the independent provider of mortality statistics in the United Kingdom, collecting data on all deaths registered in England and Wales, using *International Classification of Diseases, Tenth Revision (ICD‐10)* codes, and cause of death from the Medical Certificate of Cause of Death. Mortality follow‐up was available for all included patients up to July 31, 2021.

Secondary use of anonymized MINAP data set for research purposes is authorized under NHS research governance arrangements and further supported under section 251 of NHS act 2006 (NIGB: ECC1‐06(d)/ 2011), which allows researchers to use patient information collected within the data set for medical research without patient consent. Therefore, a formal ethical approval was not sought for this study. The study underwent formal ethical approval for the data linkages of the MINAP and ONS registries. Ethical approval was provided by the Health Research Authority, Health and Care Research Wales, and the Confidentiality Advisory Group, which is an independent body that provides expert advice on the use of confidential patient information.

### Study population

All index admissions between January 2005 and March 2019 with a primary diagnosis of AMI (comprising both ST‐elevation myocardial infarction [STEMI] and non‐STEMI but not including unstable angina) were extracted from MINAP and stratified according to the presence of RA at the time of presentation. This was conducted using *ICD‐10* codes M05 and M06, which were contained in the linked HES record, reflecting comorbidities at time of admission with index AMI. Linked HES records with *ICD‐10* codes M05 and M06 have been used in previous studies of the UK population to ascertain diagnosis of RA,^13^ with a consistent prevalence between studies of approximately 1%. Usage of *ICD‐10* code M05 for the diagnosis of seropositive RA has been shown to have a positive predictive value of 77% to 94% in administrative data sets, and usage of more detailed *ICD‐10* definitions, as we demonstrate in Supplementary Table 1, have been shown to increase this further.^14^, ^15^, ^16^

AMI diagnosis was made by clinicians according to the presenting history, clinical examination and investigations, and results of inpatient investigations in keeping with the consensus document of the Joint European Society of Cardiology and American College of Cardiology.^17^ Patients were excluded if there were missing data for variables such as in‐hospital mortality, MACE, cause of death, or missing unique identifier, which is the patient NHS number. The first admission with AMI over our study period for each patient was included, with duplicate records or readmissions over the study period identified and removed using the date of admission and NHS number.

### Outcomes

The primary outcomes were all‐cause mortality, assessed at 30 days, 1 year, 5 years, and over the entire study period, which ended in July 2021, which we referred to as “overall mortality.” Secondary outcomes of interest were quality of care measures, including the Opportunity‐Based Quality Indicators, which consists of prescription of aspirin, P2Y12 inhibitors, beta‐blockers, statins, angiotensin‐converting enzyme (ACE) inhibitors/angiotensin receptor blockers, and whether referral to cardiac rehabilitation was made while an inpatient. Additionally, we assessed CV mortality only, with a full list of *ICD‐10* codes used for assessing CV death displayed in the Supplementary Materials. One‐year mortality according to RA status was calculated for each study year to display temporal trends over the study period.

### Statistical analysis

Continuous variables such as age at admission and body mass index were summarized using mean and SD if normally distributed and median and interquartile ranges if the data were not normally distributed. Normality of distribution was assessed using the Shapiro‐Wilk test. These data were compared using Student's *t*‐test if normally distributed and Wilcoxon rank sum test if not normally distributed. Categorical variables were compared using the Pearson chi‐square test and summarized as percentages. Multiple imputation with 10 imputed data sets was used to account for the missing data across our data set after first undertaking a complete case analysis. The imputation model closely resembled the analytical model and included survival times and outcomes to avoid bias toward the null. Results of the complete case analysis undertaken before multiple imputation are shown in the Supplementary Materials. Multiple imputation with chained equations (MICE) is the best practice when dealing with missing data and can provide unbiased estimates even with high levels of missingness and some protection when data are missing not at random.^18^ Imputed data were used for our adjusted models as detailed below, whereas descriptive tables are reflective of registry data before imputation, where denominators reflect all patients for which data was available for that variable, hence the varying denominators throughout.

Multivariate Cox models were applied to 10 imputed data sets to generate adjusted hazard ratios (aHRs) with 95% confidence intervals (CIs) for mortality over our study period, with estimates combined using Rubin's rules.^19^ Our model was adjusted for the following characteristics: age, sex, ethnicity, year of admission, hospital region, heart rate, blood pressure, comorbid conditions (hypertension; diabetes mellitus; history of asthma or chronic obstructive pulmonary disease [COPD]; history of cerebrovascular accident or peripheral vascular disease; hypercholesterolemia; family history of coronary artery disease; smoking history; chronic renal failure; previous AMI; angina; previous percutaneous coronary intervention [PCI] and previous coronary artery bypass graft [CABG]; and medical therapy, including warfarin, invasive coronary angiogram, and inpatient revascularization by PCI or CABG) cardiac arrest, left ventricular systolic function, ischemic electrocardiogram change, and Killip classification. Variables included were collected at baseline, with time‐varying variables not included in this study. The HRs shown are from a comparison of patients with RA at the time of admission with AMI with those without RA. Separate Cox models were used to create HRs for 30‐day, 1‐year, 5‐year, and overall mortality (referring to until the study endpoint of July 31, 2021). Kaplan‐Meier curves were plotted to demonstrate unadjusted survival, and the ‘stcurve’ function was used in Stata 18.0 to illustrate adjusted survival, using the previously specified Cox model on a single extracted data set from our imputed model. Cox models were judged to be appropriate after assessment of Kaplan‐Meier survival curves and confirmed by the assessment of the Schoenfeld residuals, demonstrating proportional hazard over our study period.

A secondary analysis was undertaken to assess the risk of CV mortality over the study period, adjusting for the same variable list as the previous Cox model, with CV mortality as the failure and remaining non‐CV mortality censored at the time of occurrence. For the display of temporal trends of 1‐year mortality and 5‐year mortality, years were arranged into 3‐year periods to ensure adequate numbers of deaths in people with RA (2005–2007, 2008–2010, 2011–2013, 2014–2016, and 2017–2019; not included in 5‐year mortality), and the risk of mortality was calculated using our Cox regression model for each period, correcting for the same variables, aside from year of study, which was removed as a covariate.

Supplementary analyses assessed CV mortality with non‐CV mortality as a competing risk with a Fine and Gray competing risk regression model. Given our substantial population size, we used one‐to‐one nearest neighbor propensity score matching, with matching undertaken for all previously defined Cox model covariates using the ‘psmatch2’ function on Stata 18.0. A Fine and Gray competing risk regression model was then applied, with non‐CV mortality as a competing risk. Further supplementary analyses identified patients with *ICD‐10* code Z922 (personal history of drug therapy, including monoclonal drugs, regular glucocorticoids, and immune checkpoint inhibitors) and used this to form “mild” and “severe” RA groups. All statistical analyses were performed using Stata (version 18.0). The authors do not have authorization to share the data, but the data can be accessed through contacting the National Institute for CV Outcomes Research on approval.

## RESULTS

### Study population and baseline characteristics

A total of 784,091 patients with AMI remained after applying the exclusion criteria (Supplementary Figure 1): 6,047 with a diagnosis of RA at the time of admission (1%) and 778,044 without. People with RA were older in median years (74 vs 70), more likely to be female (56% vs 34%) and more likely to be of White race (94% vs 91%) (all *P* < 0.001) (Table 1).

**Table 1:** Baseline demographics of people with AMI according to presence of RA*

Variables	RA (N = 6,047)	AMI with no RA (N = 778,044)	*P* value
Age, median (IQR), y	74 (65–81)	70 (59–80)	<0.001
Female, n/N (%)	3,364/6,047 (56)	261,753/778,044 (34)	<0.001
BMI, median (IQR), kg/m^2^	26.4 (23.0–30.4)	26.9 (24.0–30.5)	<0.001
Ethnicity: White, n/N (%)	4,104/4,371 (94)	353,299/389,833 (91)	<0.001
Ethnicity: Asian, n/N (%)	169/4,371 (4)	24,046/389,833 (6)	
Ethnicity: Black, n/N (%)	40/4,371 (1)	3,918/389,833 (1)	
Ethnicity: Mixed, n/N (%)	58/4,371 (1)	8,570/389,833 (2)	
Basal crepitations, n/N (%)	698/4,080 (17)	44,132/345,458 (13)	<0.001
Pulmonary edema, n/N (%)	217/4,080 (5)	17,311/345,458 (5)	
Cardiogenic shock, n/N (%)	70/4,080 (2)	5,698/345,458 (2)	
EKG ST changes, n/N (%)	4,816/5,923 (81)	653,385/752,328 (87)	<0.001
Previous smoker, n/N (%)	2,096/5,601 (37)	239,615/720,020 (33)	<0.001
Current smoker, n/N (%)	1,173/5,601 (21)	202,349/720,020 (28)	<0.001
CCF, n/N (%)	314/5,431 (6)	37,337/704,929 (5)	0.112
Hypercholesterolemia, n/N (%)	1,388/5,396 (26)	223,191/702,101 (32)	<0.001
Cerebrovascular disease, n/N (%)	431/5,429 (8)	58,423/705,183 (8)	0.357
History of angina, n/N (%)	1,084/5,445 (20)	160,857/711,075 (23)	<0.001
Peripheral vascular disease, n/N (%)	241/5,393 (4)	30,311/699,205 (4)	0.631
Chronic renal failure, n/N (%)	378/5,423 (7)	39,463/703,966 (6)	<0.001
Diabetes mellitus, n/N (%)	1,138/5,880 (19)	156,539/751,208 (21)	0.005
Hypertension, n/N (%)	2,851/5,476 (52)	357,274/718,109 (50)	0.001
Asthma/COPD, n/N (%)	1,204/5,437 (22)	107,087/701,425 (15)	<0.001
Family history of CAD, n/N (%)	1,082/4,590 (24)	178,783/581,791 (31)	<0.001
Previous AMI, n/N (%)	809/5,463 (15)	123,930/718,248 (17)	<0.001
Previous PCI, n/N (%)	389/5,383 (7)	50,168/706,756 (7)	0.715
Previous CABG, n/N (%)	235/5,392 (4)	39,781/708,137 (6)	<0.001
STEMI, n/N (%)	2,016/6,047 (33)	303,966/778,044 (39)	<0.001
Heart rate, median (IQR), bpm	80 (69–94)	78 (66–92)	<0.001
Systolic blood pressure, median (IQR), mm Hg	137 (119–156)	138 (120–157)	0.090
Good LV function, n/N (%)	1,733/4,591 (38)	192,704/539,767 (36)	<0.001
Moderate LVSD, n/N (%)	1,052/4,591 (23)	118,860/539,767 (22)	
Severe LVSD, n/N (%)	351/4,591 (8)	39,406/539,767 (7)	
Cardiac arrest, n/N (%)	296/5,910 (5)	48,125/753,488 (6)	<0.001

* AMI, acute myocardial infarction; BMI, body mass index; CABG, coronary artery bypass graft; CAD, coronary artery disease; CCF, congestive cardiac failure; COPD, chronic obstructive pulmonary disease; EKG, electrocardiogram; IQR, interquartile range; LV, left ventricular; LVSD, LV systolic dysfunction; PCI, percutaneous coronary intervention; RA, rheumatoid arthritis; STEMI, ST‐elevation myocardial infarction.

People with RA were less likely to be current smokers (21% vs 28%), less likely to have hypercholesterolemia (26% vs 32%), and less likely to present with STEMI (33% vs 39%) (all *P* < 0.001). People with RA were more likely to have a diagnosis of asthma or COPD (22% vs 15%, *P* < 0.001) and were more likely to present with diabetes mellitus (21% vs 19%, *P* = 0.005). Supplementary Figure 2 shows the increasing proportion of patients with AMI with a diagnosis of RA in the MINAP registry over the study period, from 0.4% in 2005 to 1.7% in 2019. Supplementary Figure 3 illustrates the different proportions of cause of death between patients with RA and those without RA. People with RA were less likely to die of a CV cause (37% vs 41%) but were more likely to die because of chronic respiratory disease (11% vs 7%).

### Quality of AMI care

People with RA were less likely to receive low molecular weight heparin (LMWH) (49% vs 55%) but more likely to receive fondaparinux (42% vs 30%) (both *P* < 0.001). Rates of prescription of aspirin (96% vs 96%, *P* = 0.006) and P2Y12 inhibitors (86% vs 86%, *P* = 0.130) were similar (Table 2). People with RA were less likely to receive ACE inhibitors (71% vs 75%) or statins (80% vs 83%) post‐AMI (both *P* < 0.001). Rates of invasive coronary angiography (68% vs 68%, *P* = 0.478), PCI (43% vs 44%, *P* = 0.642), and CABG (2% vs 3%, *P* = 0.005) were similar between groups.

**Table 2:** Management strategy and clinical outcome comparison between people with AMI according to presence of RA*

Variables	RA (N = 6,047)	AMI with no RA (N = 778,044)	*P* value
Low molecular‐weight heparin, n/N (%)	2,347/4,785 (49)	356,160/645,362 (55)	<0.001
Fondaparinux, n/N (%)	1,870/4,504 (42)	165,686/544,626 (30)	<0.001
Warfarin, n/N (%)	255/4,776 (5)	33,600/634,894 (5)	0.885
Glycoprotein 2b/3a inhibitor, n/N (%)	294/4,821 (6)	56,028/647,310 (9)	<0.001
IV nitrate, n/N (%)	776/4,778 (16)	112,371/635,401 (18)	0.009
MRA, n/N (%)	326/4,181 (8)	33,964/454,171 (7)	0.435
Aspirin, n/N (%)	5,751/6,010 (96)	743,675/771,812 (96)	0.006
P2Y12 inhibitor, n/N (%)	5,100/5,916 (86)	637,060/745,000 (86)	0.130
Statins, n/N (%)	4,794/5,994 (80)	634,549/767,133 (83)	<0.001
ACE inhibitors/ARB, n/N (%)	4,268/5,973 (71)	571,091/764,507 (75)	<0.001
Beta‐blockers, n/N (%)	4,827/5,999 (80)	603,214/766,230 (79)	0.001
Inpatient invasive coronary angiogram, n/N (%)	3,946/5,800 (68)	505,413/738,159 (68)	0.478
Inpatient PCI, n/N (%)	2,578/5,962 (43)	332,127/762,804 (44)	0.642
CABG surgery, n/N (%)	102/4,378 (2)	16,787/597,557 (3)	0.055
Revascularization (CABG surgery/PCI), n/N (%)	2,662/5,921 (45)	348,182/762,988 (46)	0.299
Inpatient mortality, n/N (%)	437/6,047 (7)	45,696/778,044 (6)	<0.001
Thirty‐day mortality, n/N (%)	520/6,047 (9)	55,638/778,044 (7)	<0.001
One‐year mortality, n/N (%)	1,183/6,047 (20)	123,658/778,044 (16)	<0.001
Five‐year mortality (Kaplan‐Meier estimate), %	45	33	
Reinfarction, n/N (%)	65/5,461 (1)	10,167/700,668 (1)	0.108
Major bleeding, n/N (%)	49/5,835 (1)	6,522/747,931 (1)	0.792
MACE, n/N (%)	484/6,047 (8)	53,760/778,044 (7)	0.001

* MACE is defined as the composite endpoint of in‐hospital death and reinfarction. ACE, angiotensin‐converting enzyme; AMI, acute myocardial infarction; ARB, angiotensin receptor blocker; CABG, coronary artery bypass graft; IV, intravenous; MACE, major adverse cardiovascular event; MRA, mineralocorticoid receptor antagonist; PCI, percutaneous coronary intervention; RA, rheumatoid arthritis.

### Post‐AMI outcomes

**Figure 1:**
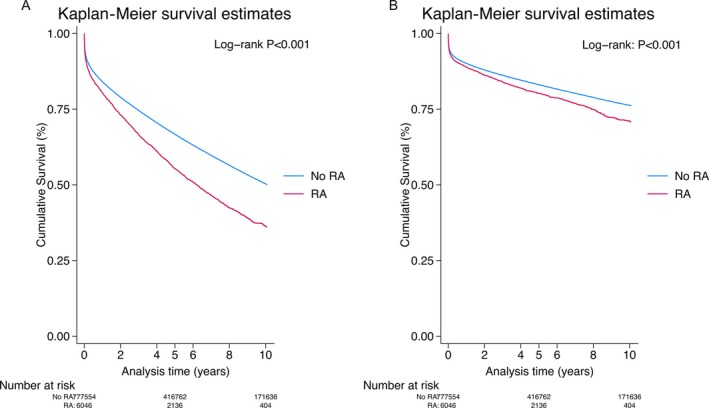
Kaplan‐Meier survival analysis for patients with acute myocardial infarction and RA compared with those without (A) all‐cause mortality or (B) cardiovascular mortality. RA, rheumatoid arthritis.

Inpatient (7% vs 6%), 30‐day (9% vs 7%), and 1‐year (20% vs 16%) all‐cause mortality were higher in the RA cohort (all *P* < 0.001). The Kaplan‐Meier estimate of 5‐year all‐cause mortality was significantly higher in the RA cohort (45% vs 33%). Figure 1A illustrates the long‐term unadjusted survival of people with RA compared with people with >10 years of follow‐up where available. Figure 1B illustrates long‐term unadjusted survival from CV causes of death only over the same study period. Supplementary Figure 4 demonstrates a log plot of our survival model, and Supplementary Figure 5 shows variables included in our multiple imputation model. Supplementary Figure 6 shows adjusted survival over our entire study period.

**Figure 2:**
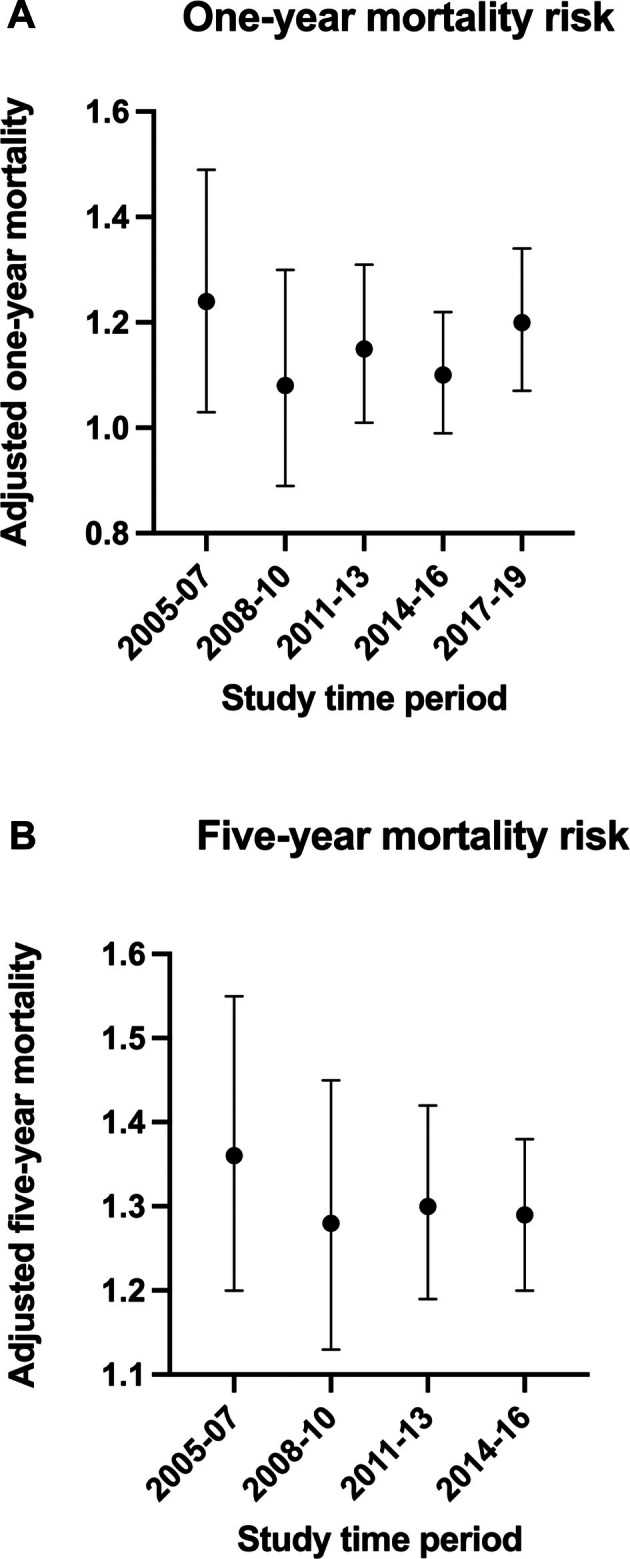
Temporal trends of all‐cause mortality over the study period according to the presence of rheumatoid arthritis. (A) One‐year mortality. (B) Five‐year mortality.

There was no significant difference in 30‐day mortality between people with RA and those without after the models were adjusted for all available confounders (aHR 1.0, 95% CI 0.99–1.19; *P* = 0.075) (Table 3). However, at 1 year (aHR 1.14, 95% CI 1.07–1.21; *P* < 0.001), 5 years (aHR 1.28, 95% CI 1.23–1.33; *P* < 0.001), and to the end of the study period (July 31, 2021) (aHR 1.31, 95% CI 1.26–1.36; *P* < 0.001), the risk of all‐cause mortality was significantly higher in the RA cohort. Thirty‐day (aHR 1.08, 95% CI 0.97–1.19; *P* = 0.172) and 1‐year (aHR 1.08, 95% CI 1.00–1.17; *P* = 0.058) CV‐specific mortality was not significantly higher in those with RA, but 5‐year (aHR 1.15, 95% CI 1.08–1.23; *P* < 0.001) and total study period CV mortality (aHR 1.18, 95% CI 1.11–1.24; *P* < 0.001) were higher in those with RA (Table 4). Figure 2A and B illustrates the temporal trends of adjusted 1‐year and 5‐year mortality for the five 3‐year periods across our study period, illustrating that the disparity in 1‐year and 5‐year mortality across study years according to the presence of RA has not improved in the years between 2005 and 2019.

**Table 3:** All‐cause mortality survival analysis for people with acute myocardial infarction with or without RA at time of presentation*

Outcome variables	aHR (95% CI)^a^	*P* value
Primary outcomes		
Thirty‐day mortality	1.09 (0.99–1.19)	0.075
One‐year mortality	1.14 (1.07–1.21)	<0.001
Five‐year mortality	1.28 (1.23–1.33)	<0.001
Overall mortality	1.31 (1.26–1.36)	<0.001

* The aHRs are presented with 95% CIs, adjusted for age at admission, sex, ethnicity, year of admission, heart rate, blood pressure, comorbid conditions (hypertension; diabetes mellitus; history of asthma or chronic obstructive pulmonary disease; history of cerebrovascular accident or peripheral vascular disease; hypercholesterolemia; family history of coronary artery disease; smoking history; chronic renal failure; previous acute myocardial infarction; angina; previous percutaneous coronary intervention and previous coronary artery bypass graft; and medical therapy, including warfarin, invasive coronary angiogram, and inpatient revascularization by percutaneous coronary intervention or coronary artery bypass graft), cardiac arrest, left ventricular systolic function, Killip classification, and admission hospital region. aHR, adjusted hazard ratio; CI, confidence interval; RA, rheumatoid arthritis.

^a^ Values show comparison between patients with RA and those without.

**Table 4:** Cardiovascular mortality survival analysis for people with acute myocardial infarction with or without RA at time of presentation*

Outcome variables	aHR (95% CI)^a^	*P* value
Primary outcomes		
Thirty‐day mortality	1.06 (0.95–1.17)	0.322
One‐year mortality	1.08 (1.00–1.17)	0.058
Five‐year mortality	1.15 (1.08–1.23)	<0.001
Overall mortality	1.18 (1.11–1.24)	<0.001

* The aHRs are presented with 95% CIs, adjusted for age at admission, sex, ethnicity, year of admission, heart rate, blood pressure, comorbid conditions (hypertension; diabetes mellitus; history of asthma or chronic obstructive pulmonary disease; history of cerebrovascular accident or peripheral vascular disease; hypercholesterolemia; family history of coronary artery disease; smoking history; chronic renal failure; previous acute myocardial infarction; angina; previous percutaneous coronary intervention and previous coronary artery bypass graft; and medical therapy, including warfarin, invasive coronary angiogram, and inpatient revascularization by percutaneous coronary intervention or coronary artery bypass graft), cardiac arrest, left ventricular systolic function, Killip classification, and admission hospital region. Noncardiovascular mortality was censored at the time of occurrence. aHR, adjusted hazard ratio; CI, confidence interval; RA, rheumatoid arthritis.

^a^ Values show comparison between patients with RA and those without.

### Supplementary analyses

Supplementary Table 1 shows the *ICD‐10* diagnostic codes used to identify patients with RA. When evaluating specific quality indicators for patients without STEMI, the proportion of people undergoing their invasive coronary angiogram within 72 hours was not significantly different between people with or without RA (58% vs 57%, *P* = 0.813) (Supplementary Table 2). The proportion of people receiving dual antiplatelet therapy (DAPT) was the same between groups (83% vs 83%, *P* = 0.605). The proportion of people referred to cardiac rehabilitation on discharge was lower in the RA group (76% vs 78%, *P* < 0.001).

For STEMI, people with RA had a lower proportion of patients receiving a call to balloon time of <120 minutes (51% vs 60%, *P* < 0.001), although there was no significant difference in the proportion of people achieving a door to balloon time of <60 minutes (71% vs 73%, *P* = 0.149) or 90 minutes (85% vs 86%, *P* = 0.108) (Supplementary Table 3). People with RA were more likely to have their left ventricular function assessed while an inpatient (76% vs 71%, *P* < 0.001). There was no significant difference in mean Opportunity‐Based Quality Indicators score between people with and without RA (86.9 vs 87.0, *P* = 0.086). The results of our complete case analysis, on all study participants with full data available are shown in Supplementary Tables 4–6.

The results for our supplementary analysis, categorizing patients’ severity of RA (mild or severe) according to whether patients are taking disease‐modifying antirheumatic drugs or biologic therapies are shown in Supplementary Table 7 (all‐cause mortality) and Supplementary Table 8 (CV mortality). When including non‐CV mortality as a competing risk, the results for CV mortality were very similar, with no significant increase in risk at 1 year (aHR 1.04, 95% CI 0.93–1.17; *P* = 0.478) but elevated risk in people with RA at 5 years (aHR 1.11, 95% CI 1.01–1.22; *P* = 0.029) and for the total study period (aHR 1.10, 95% CI 1.01–1.21; *P* = 0.025) (Supplementary Table 9).

## DISCUSSION

Our comprehensive analysis of >780,000 individuals admitted with AMI, with a median follow‐up of >6 years, shows important differences in the long‐term outcomes of people with RA and AMI. First, the number of people in our registry with RA presenting with AMI increased progressively over our study period, likely reflecting that this is a growing population, worthy of future study and additionally reflecting improved comorbidity coding over the study. Second, there was no significant difference in the quality of care received between people with or without RA after AMI, with people with RA receiving high‐quality AMI care throughout the course of the study, with regard to guideline‐directed medical therapy, invasive coronary angiography, and revascularization by either PCI or CABG.

Despite this, people with RA had significantly higher adjusted all‐cause mortality at 1 year, 5 years, and up to the end of the study period, with a median follow‐up of 6 years for included persons. Importantly, this elevated mortality risk was not significant at 30 days. People with RA had significantly higher CV mortality at 1 year and beyond, emphasizing the importance of CVD in the RA cohort. Despite contemporary improvements in therapies for RA, the increased risk of 1‐year mortality for patients with RA remains persistent from the early to latter years of our study.

Previous studies in this area have important limitations, meaning that our study adds an important perspective in the contemporary management of AMI in people with RA in a large, universal health care system. Studies have frequently been limited by either a small population of people with RA, who are limited to either in‐hospital or short‐term mortality follow‐up, or have mostly enrolled patients in the early 2000s, when access to newer biologic therapies was more restricted.^6^, ^17^

Given that people with RA have a systemic inflammatory response, leading to significantly higher concentrations of circulating pro‐inflammatory cytokines, it is no surprise that there is accelerated formation of coronary artery plaque^4^ alongside a challenging combination of endothelial dysfunction, insulin resistance, and dyslipidemia, which all contribute to the excess CV mortality seen in those with RA.^4^, ^5^ The long‐term all‐cause mortality risk in those with RA after AMI is replicated in studies worldwide, in the United States, Finland, Sweden, and Taiwan,^6^, ^7^, ^20^, ^21^ for example showing a consistent association with premature mortality across different health care systems and populations. Our study adds to the literature that in individuals with AMI those with RA have a higher risk of long‐term all‐cause mortality, but we additionally demonstrate the importance of CV‐specific mortality.

Although the picture of elevated long‐term all‐cause mortality is now clear, there has been uncertainty in the interpretation of literature regarding in‐hospital quality of care and early mortality according to the presence of RA. Previous studies have shown a range of findings in this domain. Van Doornum et al demonstrated poorer rates of guideline‐directed medical therapy, reperfusion, and in‐hospital mortality in those with RA,^9^ and Lai et al noted increased in‐hospital and overall mortality in patients with RA in their nationwide Taiwanese study.^21^ Conversely, Francis et al and Elbadawi et al show that RA was associated with lower inpatient mortality in their US nationwide studies.^8^, ^22^ With no significant difference in in‐hospital quality of care or all‐cause mortality at 30 days in our study, we add further evidence to the suggestion that in a contemporary, universal health care setting, there is no significant disparity in access to high‐quality AMI care for people with RA and that their early mortality post‐AMI does not seem to be any different to that of the non‐AMI population.

Although we are considering the impact of AMI, it must be considered that people with RA already have significantly elevated longer‐term all‐cause mortality than the general population, with higher rates of not just MI but additionally heart failure and stroke.^23^ This is not surprising, given the elevated risk of important modifiable risk factors such as obesity, physical inactivity, hypertension, hyperlipidemia, and diabetes mellitus in people with RA, which are significant contributors to the elevated long‐term all‐cause mortality seen in this cohort.^24^, ^25^, ^26^ Furthermore, there is increased risk associated with medications used to treat RA, such as glucocorticoids and JAK inhibitors, with regard to CVD and all‐cause mortality.^27^, ^28^ Historically, it has been the excess burden of CV mortality that is a major driver of this higher all‐cause mortality, but there has been recent evidence suggesting that CV mortality in patients with RA is declining, likely through a combination of improvements in RA therapy and reductions in modifiable risk factors such as smoking, whereas respiratory and neoplastic causes of death are either static or increasing.^29^ There are also growing data in the more contemporary era to suggest that in well‐treated inflammatory arthritis, 5‐year survival is becoming comparable with that of the baseline population.^30^ It was therefore disappointing that despite advances in RA therapy over our study period, this has not translated to narrowing the disparity in survival post‐AMI, and that CV‐specific mortality remains a significant contributor to this. Our cause of death analysis on our RA population shows higher rates of neoplastic, chronic respiratory and infective deaths than the general AMI population, which is in keeping with previous analyses of cause of death in people with RA.^31^

One area of potential improvement in the quality of care of all patients post‐AMI could be increased rates of referral to cardiac rehabilitation at the point of discharge, an intervention with well‐established evidence to reduce long‐term mortality and improve quality of life.^32^ We acknowledge that there could be patient‐level reasons why referral, attendance, or completion of cardiac rehabilitation is lower than ideal in all patients and lower still in patients with RA. Furthermore, our study shows that over time, the proportion of people with an RA diagnosis at the time of AMI is increasing. This is in keeping with predictive modeling using data from the Global Burden of Diseases, Injuries, and Risk Factors Study suggesting that the RA population will continue to grow over the coming years, partly attributable to improvement of diagnosis and reduction of excess mortality in early RA.^33^, ^34^ We suspect that improvement in the comorbidity coding for AMI admissions in the MINAP registry also contributes. Despite this, we suggest that the rates of people with RA presenting with AMI are increasing, just not to the extent suggested by our temporal trends analysis.

In summary, our findings indicate that RA remains associated with significantly increased long‐term mortality following AMI, even in the context of equitable inpatient care, and that this disparity has not narrowed over a 15‐year study period despite advances in modern RA therapies. We suggest that further studies should focus on identifying and addressing the disparities in the management of RA and its effects on patients’ long‐term health and to determine if more aggressive RA therapies are warranted.

There are several major strengths to this study. The MINAP registry collects robust data, with many variables recorded from all those presenting to the hospital with AMI in the United Kingdom. Our results are more likely to be representative of other publicly funded health care models globally, owing to the balancing out of regional differences. Our postdischarge mortality data from the ONS enables us to assess cause‐specific mortality at least 2 years for all included persons. Furthermore, our paper studies people within a universal health care system, in which health care is free at the point of use; therefore, there should not be significant regional or socioeconomic disparities in access to aspects of high‐quality AMI care that one may expect to see in insurance‐based health care systems.^35^

There are several important limitations to consider in this study. First, there is no external validation of data inputs in the MINAP database. RA status is determined by HES coding, which may lack clinical verification (eg, seropositive vs seronegative RA). We can see increased and improved comorbidity coding over the study period; therefore, we suspect that the proportion of people with RA in the early years of the study is underestimated. Second, although the MINAP database collects many variables, it does not collect data on frailty, an exhaustive list of comorbidities, disease severity, or the intensity or timescale of RA treatments; these important covariates are not then able to be included within our adjusted models, leaving a risk of residual confounding. Therefore, our ability to subclassify patients is limited and we may miss disparities in management. We additionally lack detailed data on baseline CV risk status of patients, with no access to whether there is baseline coronary calcification from prior imaging, for example, and we do not have access to biomarker data such as high‐sensitivity C‐reactive protein levels. Furthermore, long‐term mortality outcomes may be influenced by adherence to therapy, ongoing RA care, or postdischarge cardiac follow‐up, none of which are captured, and therefore cannot be included within our adjusted models.

Given that we are assessing long‐term mortality, a further limitation is our usage of baseline variable collection at time of admission, and we are unable to adjust for time‐varying confounders over the course of the study. The most important limitation of our study is the lack of ability with which to accurately stratify the severity of RA given the limited capture of important additional metrics that could stratify severity, such as the usage of disease‐modifying antirheumatic drugs, usage of long‐term glucocorticoids, or active usage of biologic therapy. Additionally, the inclusion of a second measure of RA status would improve the reliability of capturing RA status from administrative data sets, and we acknowledge a risk of misclassification of patients because of the lack of a second measure.^36^ Overall, our method of identification of patients with RA carries a risk of overestimating patient numbers by including patients that either do not have RA or have very mild disease; therefore, we may be underestimating mortality risk post‐AMI in the RA cohort by including low‐risk patients, reflecting bias toward the null, and it is important that our results are interpreted in this context.

There was a possible selection bias in our quality‐of‐care groups, because to receive a referral for cardiac rehabilitation people need to be fit enough to attend, have sufficient renal function to tolerate ACE inhibitors, and have a prognosis good enough to start on statins. Finally, it must be considered that MINAP is an AMI‐specific registry, and we are unable to compare outcomes with people without AMI.

Our nationwide analysis of the long‐term outcomes of >780,000 people in England and Wales with AMI, 6,047 of whom had RA, shows that there is a growing proportion of patients admitted with AMI who have a diagnosis of RA, but those with RA do not experience significant disparities in inpatient AMI care. Although there is no significant difference in 30‐day mortality, those with RA have an elevated risk of long‐term all‐cause and CV‐specific mortality. Finally, we report that the degree of elevated mortality risk in those with RA has not decreased over our study period despite advances in RA therapies.

## AUTHOR CONTRIBUTIONS

All authors contributed to at least one of the following manuscript preparation roles: conceptualization AND/OR methodology, software, investigation, formal analysis, data curation, visualization, and validation AND drafting or reviewing/editing the final draft. As corresponding author, Professor Mamas confirms that all authors have provided the final approval of the version to be published, and takes responsibility for the affirmations regarding article submission (eg, not under consideration by another journal), the integrity of the data presented, and the statements regarding compliance with institutional review board/Declaration of Helsinki requirements.

## Supporting information


**Disclosure Form**:

**Data S1** Supporting Information
