# Supplementary material for: Temporal Trends and Short‐ and Long‐Term Mortality of People With Acute Myocardial Infarction and Rheumatoid Arthritis: A Nationwide Cohort Study
Source: Arthritis Care Res (Hoboken). 2026 Mar 7;78(7):850–9. doi: 10.1002/acr.70009 (PMC13313107; doi:10.1002/acr.70009)
Supplement: Supplementary file 2 — Data S1 Supporting Information [file ACR-78-850-s002.docx]

**Supplementary Table 1: ICD-10 codes used to extract rheumatoid arthritis cohort**

| **ICD-10 Codes used** |
| --- |
| **M05:** Seropositive rheumatoid arthritis |
| **M05.1:** Rheumatoid lung disease |
| **M05.2:** Rheumatoid vasculitis |
| **M05.3:** Rheumatoid arthritis with involvement of other organs and systems |
| **M05.8:** Other seropositive rheumatoid arthritis |
| **M05.9:** Seropositive rheumatoid arthritis |
| **M06:** Other rheumatoid arthritis |
| **M06.0:** Seronegative rheumatoid arthritis |
| **M06.2:** Rheumatoid bursitis |
| **M06.3:** Rheumatoid nodule |
| **M06.8:** Other specified rheumatoid arthritis |
| **M06.9:** Rheumatoid arthritis, unspecified |

**Supplementary Table 2: Quality Indicators for people with NSTEMI according to presence of rheumatoid arthritis (ESC ACVC and OBQI)**

|  | **NSTEMI and Rheumatoid arthritis (RA) (n=4,031)** | **NSTEMI with no RA (n=474,078)** | **P-value** |
| --- | --- | --- | --- |
| Coronary Angiography received within 72 hours (%) | 930/1,615 (58) | 102,724/179,299 (57) | 0.813 |
| LV Function recorded in notes (%) | 2,007/3,097 (65) | 201,852/329,761 (61) | <0.001 |
| Fondaparinux or LMWH received (%) | 2,827/3,299 (86) | 348,971/396,029 (88) | <0.001 |
| DAPT received on discharge (%) | 3,291/3,964 (83) | 378,249/457,319 (83) | 0.605 |
| ACEi or ARB on discharge for those with moderate and severe LVSD (%) | 567/769 (74) | 59,640/78,466 (76) | 0.142 |
| Beta Blocker on discharge for those for those with moderate and severe LVSD (%) | 662/771 (86) | 64,413/78,628 (82) | 0.005 |
| Composite All/None score* (%) | 2,656/3,956 (67) | 317,979/457,689 (69) | 0.001 |
| Composite All/None score for those with moderate and severe LVSD (%) | 542/769 (71) | 57,193/78,294 (73) | 0.110 |
| Mean OBQI score | 81.9 | 82.7 | 0.014 |
| Cardiac rehabilitation (%) | 2,780/3,655 (76) | 334,067/427,848 (78) | <0.001 |

ESC; European Society of Cardiology, ACVC; Association for Acute Cardiovascular Care, GRACE; global registry of acute coronary events, CRUSADE; can rapid risk stratification of unstable angina patients suppress adverse outcomes with early implementation of the ACC/AHA guidelines, LV; left ventricle, EF; ejection fraction, LMWH; low molecular weight heparin, DAPT; dual antiplatelet therapy, ACEi/ARB; angiotensin converting enzyme inhibitor/angiotensin receptor blockers, LVSD; left ventricular systolic dysfunction, N/A; Not Available.

*Composite score of receipt of low dose aspirin, P2Y_12_ inhibition and statin.

**Opportunity based QI (The score consisted of 6 evidence-based processes of care: the prescription of aspirin, thienopyridine inhibitor, β-blocker, angiotensin converting enzyme inhibitor (ACEi), HMG CoA reductase enzyme inhibitor (statin) and enrolment onto a cardiac rehabilitation programme at the time of discharge). The OBCS reflects the number of care opportunities fulfilled at each hospital (numerator) divided by the number of opportunities to provide care (denominator). Excluded from both numerator and denominator were particular interventions that were contra-indicated, not applicable, not indicated in, or declined by, individual patients.

**Supplementary Table 3: Quality Indicators for people with STEMI according to presence of rheumatoid arthritis (ESC ACVC and OBQI)**

| **Variables** | **STEMI and Rheumatoid arthritis (RA) (n=2,016)** | **STEMI with no RA (n=303,966)** | **P-value** |
| --- | --- | --- | --- |
| Reperfusion within 12 h of presentation | 1,371/1,394 (98) | 222,258/225,489 (99) | 0.597 |
| Door-to-balloon time <60 min | 992/1,394 (71) | 164,352/225,489 (73) | 0.149 |
| Door-to-balloon time <90 min | 1,185/1,394 (85) | 195,016/225,489 (86) | 0.108 |
| Call to balloon time <120 min | 651/1,274 (51) | 121,555/201,070 (60) | <0.001 |
| Revascularization (PCI/CABG) | 1,406/1,989 (71) | 202,522/299,233 (68) | 0.004 |
| Left ventricular ejection fraction assessed | 1,129/1,494 (76) | 149,118/210,006 (71) | <0.001 |
| DAPT received on discharge | 1,651/1,953 (85) | 243,023/289,124 (84) | 0.562 |
| ACE inhibitor or ARB on discharge for those with moderate and severe LVSD (%) | 512/630 (81) | 67,166/79,205 (85) | 0.014 |
| Mean OBQI score | 86.9 | 87.0 | 0.086 |
| Cardiac rehabilitation (%) | 1,527/1,804 (85) | 233,128/270,876 (86) | 0.004 |

Data are expressed as proportions (%) unless indicated otherwise. Denominators represent the total number of participants with a data point collected; numerators represent the number of those participants for whom the variable of interest was present

Opportunity-based care score. The score consisted of six evidence-based processes of care: prescription of aspirin, a thienopyridine inhibitor, a β-blocker, an ACE inhibitor and a hydroxymethylglutaryl-coenxyme A (HMG CoA) reductase enzyme inhibitor (statin) and enrolment onto a cardiac rehabilitation programme at the time of discharge. The score reflects the number of care opportunities fulfilled at each hospital (numerator) divided by the number of opportunities to provide care (denominator). Interventions that were contraindicated, not applicable or not indicated in or declined by individual participants were excluded from both the numerator and the denominator

**Supplementary Table 4: All-cause mortality survival analysis for people with AMI with or without rheumatoid arthritis at time of presentation (complete case analysis)**

| **Outcome variables** | **Adjusted hazard ratio for people with RA compared those without (95% CIs)** | **P-value** |
| --- | --- | --- |
| **Primary Outcomes** | | |
| **Thirty-day mortality** | 1.01 (0.85-1.20) | 0.087 |
| **One-year mortality** | 1.13 (1.01-1.25) | 0.026 |
| **Five-year mortality** | 1.31 (1.23-1.41) | <0.001 |
| **Overall mortality** | 1.33 (1.24-1.42) | <0.001 |

Adjusted Hazard ratios are presented with 95% CIs, adjusted for: age at admission, sex, ethnicity, year of admission, heart rate, blood pressure, co-morbid conditions (hypertension, diabetes mellitus, history of asthma or COPD, history of CVA or PVD, hypercholesterolaemia, family history of coronary artery disease, smoking history, chronic renal failure, previous AMI, angina, previous PCI and previous CABG, warfarin therapy, invasive coronary angiogram, inpatient revascularisation by PCI or CABG), cardiac arrest, LV systolic function, Killip classification, ischaemic ECG change, and admission hospital.

**Supplementary Table 5: Cardiovascular mortality survival analysis for people with AMI with or without rheumatoid arthritis at time of presentation (complete case analysis)**

| **Outcome variables** | **Adjusted hazard ratio for people with RA compared those without (95% CIs)** | **P-value** |
| --- | --- | --- |
| **Primary Outcomes** | | |
| **Thirty-day mortality** | 1.07 (0.88-1.30) | 0.479 |
| **One-year mortality** | 1.07 (0.92-1.23) | 0.385 |
| **Five-year mortality** | 1.16 (1.03-1.30) | 0.012 |
| **Overall mortality** | 1.17 (1.05-1.30) | 0.005 |

Adjusted Hazard ratios are presented with 95% CIs, adjusted for: age at admission, sex, ethnicity, year of admission, heart rate, blood pressure, co-morbid conditions (hypertension, diabetes mellitus, history of asthma or COPD, history of CVA or PVD, hypercholesterolaemia, family history of coronary artery disease, smoking history, chronic renal failure, previous AMI, angina, previous PCI and previous CABG, warfarin therapy, invasive coronary angiogram, inpatient revascularisation by PCI or CABG), cardiac arrest, LV systolic function, Killip classification, ischaemic ECG change, and admission hospital. Non-CV mortality censored at time of occurrence.

**Supplementary Table 6: Cardiovascular mortality survival analysis for people with AMI with or without rheumatoid arthritis at time of presentation (complete case analysis), with one-to-one propensity score matching and non-CV mortality as competing risk**

| **Outcome variables** | **Adjusted sub-hazard ratio for people with RA compared those without (95% CIs)** | **P-value** |
| --- | --- | --- |
| **Primary Outcomes** | | |
| **Thirty-day mortality** | 1.07 (0.79-1.44) | 0.644 |
| **One-year mortality** | 1.00 (0.82-1.25) | 0.978 |
| **Five-year mortality** | 1.04 (0.88-1.24) | 0.624 |
| **Overall mortality** | 1.04 (0.88-1.22) | 0.661 |

Model applied to one-to-one matched populations with propensity score matching, matched for all model covariates. Adjusted sub-hazard ratios are presented with 95% CIs, adjusted for: age at admission, sex, ethnicity, year of admission, heart rate, blood pressure, co-morbid conditions (hypertension, diabetes mellitus, history of asthma or COPD, history of CVA or PVD, hypercholesterolaemia, family history of coronary artery disease, smoking history, chronic renal failure, previous AMI, angina, previous PCI and previous CABG, warfarin therapy, invasive coronary angiogram, inpatient revascularisation by PCI or CABG), cardiac arrest, LV systolic function, Killip classification, ischaemic ECG change, and admission hospital. Non-CV mortality set as competing risk in Fine and Gray competing risk regression model.

**Supplementary Table 7: All-cause mortality survival analysis for people with AMI with or without rheumatoid arthritis at time of presentation**

| **Outcome variables** | **Adjusted hazard ratio for people with RA compared those without (95% CIs)** | | | | | | |
| --- | --- | --- | --- | --- | --- | --- | --- |
|  | **“Mild” RA** | **P-value** | | **“Severe” RA** | | | **P-value** |
| **Thirty-day mortality** | 1.12 (1.02-1.23) | | 0.020 | | 0.69 (0.45-1.04) | 0.079 | |
| **One-year mortality** | 1.15 (1.08-1.22) | | <0.001 | | 1.01 (0.81-1.27) | 0.900 | |
| **Five-year mortality** | 1.29 (1.23-1.34) | | <0.001 | | 1.21 (1.05-1.39) | 0.010 | |
| **Overall mortality** | 1.31 (1.26-1.37) | | <0.001 | | 1.25 (1.10-1.42) | 0.001mi | |

Adjusted Hazard ratios are presented with 95% CIs, adjusted for: age at admission, sex, ethnicity, year of admission, heart rate, blood pressure, co-morbid conditions (hypertension, diabetes mellitus, history of asthma or COPD, history of CVA or PVD, hypercholesterolaemia, family history of coronary artery disease, smoking history, chronic renal failure, previous AMI, angina, previous PCI and previous CABG, warfarin therapy, invasive coronary angiogram, inpatient revascularisation by PCI or CABG), cardiac arrest, LV systolic function, Killip classification, ischaemic ECG change, and admission hospital.

Mild RA refers to patients that are not recorded to be taking DMARDs, long-term corticosteroids or immunotherapy, “severe” RA patients are taking one of these agents at time of presentation.

**Supplementary Table 8: Cardiovascular mortality only survival analysis for people with AMI with or without rheumatoid arthritis at time of presentation**

| **Outcome variables** | **Adjusted hazard ratio for people with RA compared those without (95% CIs)** | | | | | | |
| --- | --- | --- | --- | --- | --- | --- | --- |
|  | **“Mild” RA** | **P-value** | | **“Severe” RA** | | | **P-value** |
| **Thirty-day mortality** | 1.08 (0.97-1.21) | | 0.156 | | 0.71 (0.44-1.14) | 0.154 | |
| **One-year mortality** | 1.09 (1.00-1.18) | | 0.046 | | 0.99 (0.74-1.34) | 0.960 | |
| **Five-year mortality** | 1.16 (1.08-1.24) | | <0.001 | | 1.07 (0.85-1.34) | 0.579 | |
| **Overall mortality** | 1.19 (1.12-1.26) | | <0.001 | | 1.10 (0.89-1.37) | 0.360 | |

Adjusted Hazard ratios are presented with 95% CIs, adjusted for: age at admission, sex, ethnicity, year of admission, heart rate, blood pressure, co-morbid conditions (hypertension, diabetes mellitus, history of asthma or COPD, history of CVA or PVD, hypercholesterolaemia, family history of coronary artery disease, smoking history, chronic renal failure, previous AMI, angina, previous PCI and previous CABG, warfarin therapy, invasive coronary angiogram, inpatient revascularisation by PCI or CABG), cardiac arrest, LV systolic function, Killip classification, ischaemic ECG change, and admission hospital.

Mild RA refers to patients that are not recorded to be taking DMARDs, long-term corticosteroids or immunotherapy, “severe” RA patients are taking one of these agents at time of presentation.

**Supplementary Table 9: Cardiovascular mortality survival analysis for people with AMI with or without rheumatoid arthritis at time of presentation, with one-to-one propensity score matching and non-CV mortality as competing risk**

| **Outcome variables** | **Adjusted sub-hazard ratio for people with RA compared those without (95% CIs)** | **P-value** |
| --- | --- | --- |
| **Primary Outcomes** | | |
| **Thirty-day mortality** | 1.04 (0.89-1.21) | 0.598 |
| **One-year mortality** | 1.04 (0.93-1.17) | 0.478 |
| **Five-year mortality** | 1.11 (1.01-1.22) | 0.029 |
| **Overall mortality** | 1.10 (1.01.1.21) | 0.025 |

Model applied to one-to-one matched populations with propensity score matching, matched for all model covariates. Adjusted sub-hazard ratios are presented with 95% CIs, adjusted for: age at admission, sex, ethnicity, year of admission, heart rate, blood pressure, co-morbid conditions (hypertension, diabetes mellitus, history of asthma or COPD, history of CVA or PVD, hypercholesterolaemia, family history of coronary artery disease, smoking history, chronic renal failure, previous AMI, angina, previous PCI and previous CABG, warfarin therapy, invasive coronary angiogram, inpatient revascularisation by PCI or CABG), cardiac arrest, LV systolic function, Killip classification, ischaemic ECG change, and admission hospital. Non-CV mortality set as competing risk in Fine and Gray competing risk regression model.

**Supplementary Figure 1: STROBE Diagram detailing study inclusion and exclusion criteria**

N=10,690,541 starting MINAP-HES episodes from 2005-2019

Missing cause of death (n=57,838)

Inconsistent HES and MINAP mortality dates (n=8,881)

Duplicate episodes according to NHS number and MINAP admission (n=6,629,132)

Total AMI patients for inclusion n=784,091

No Rheumatoid arthritis (n=778,044)

Rheumatoid arthritis (n=6.047)

**Supplementary Figure 2: Proportion of people with AMI with rheumatoid arthritis diagnosis at time of admission**

MINAP data collection is up to March 31^st^ in 2019 only.

**Supplementary Figure 3: Cause of death analysis for people with and without rheumatoid arthritis at time of admission**

1. **Cause of death for people with rheumatoid arthritis**

1. **Cause of death for people without rheumatoid arthritis**

**Supplementary Figure 4: Scaled Schoenfeld residual plot for proportional hazard model**

**
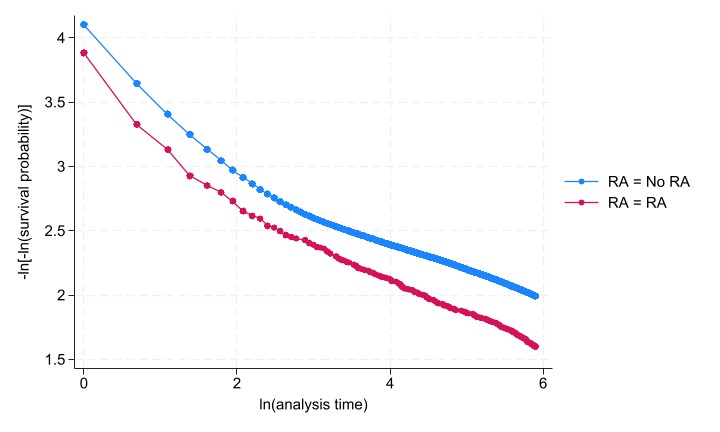
**

**Supplementary Figure 5: Multiple imputation with chained equations (MICE) model output for AMI cohort with and without RA**

**
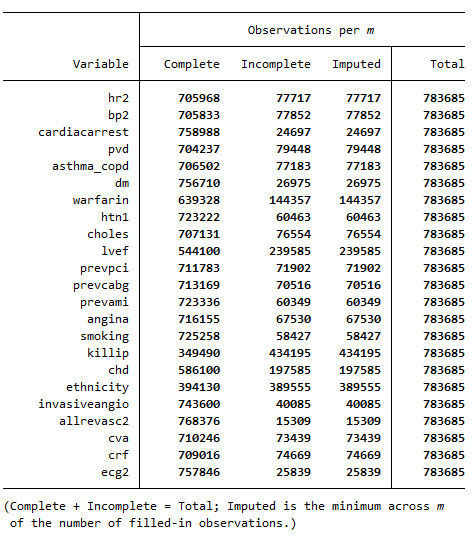
**

**Observations per m refers to observations per completed dataset within the imputed model.**

**Supplementary Figure 6: Adjusted all-cause survival according to presence of rheumatoid arthritis**

**
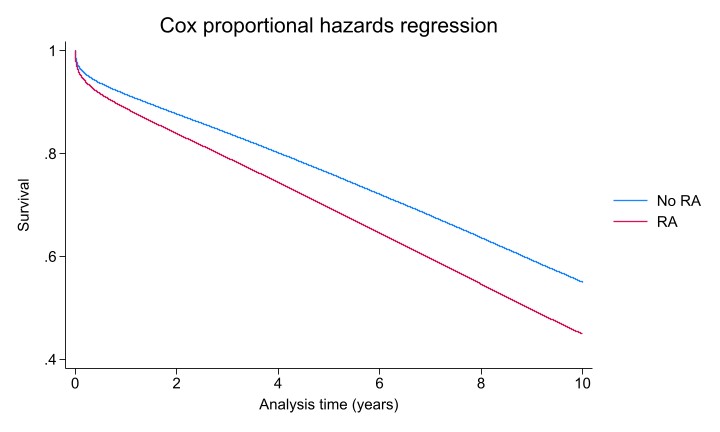
**

**Adjusted all-cause survival plotted on Stata 18.0 using Stcurve function, using the Cox-regression model, adjusted for the covariates listed in the methods.**
